# Use of Caval Subtraction 2D Phase-Contrast MR Imaging to Measure Total Liver and Hepatic Arterial Blood Flow: Preclinical Validation and Initial Clinical Translation

**DOI:** 10.1148/radiol.2016151832

**Published:** 2016-05-12

**Authors:** Manil D. Chouhan, Rajeshwar P. Mookerjee, Alan Bainbridge, Simon Walker-Samuel, Nathan Davies, Steve Halligan, Mark F. Lythgoe, Stuart A. Taylor

**Affiliations:** From the University College London Centre for Medical Imaging (M.D.C., S.H., S.A.T.), Institute for Liver and Digestive Health (R.P.M., N.D.), and Centre for Advanced Biomedical Imaging (S.W.S., M.F.L.), Division of Medicine, University College London, 250 Euston Rd, 3rd Floor East, London NW1 2PG, England; and Department of Medical Physics, University College London Hospitals NHS Trust, London, England (A.B.).

## Abstract

**Purpose:**

To validate caval subtraction two-dimensional (2D) phase-contrast magnetic resonance (MR) imaging measurements of total liver blood flow (TLBF) and hepatic arterial fraction in an animal model and evaluate consistency and reproducibility in humans.

**Materials and Methods:**

Approval from the institutional ethical committee for animal care and research ethics was obtained. Fifteen Sprague-Dawley rats underwent 2D phase-contrast MR imaging of the portal vein (PV) and infrahepatic and suprahepatic inferior vena cava (IVC). TLBF and hepatic arterial flow were estimated by subtracting infrahepatic from suprahepatic IVC flow and PV flow from estimated TLBF, respectively. Direct PV transit-time ultrasonography (US) and fluorescent microsphere measurements of hepatic arterial fraction were the standards of reference. Thereafter, consistency of caval subtraction phase-contrast MR imaging–derived TLBF and hepatic arterial flow was assessed in 13 volunteers (mean age, 28.3 years ± 1.4) against directly measured phase-contrast MR imaging PV and proper hepatic arterial inflow; reproducibility was measured after 7 days. Bland-Altman analysis of agreement and coefficient of variation comparisons were undertaken.

**Results:**

There was good agreement between PV flow measured with phase-contrast MR imaging and that measured with transit-time US (mean difference, −3.5 mL/min/100 g; 95% limits of agreement [LOA], ±61.3 mL/min/100 g). Hepatic arterial fraction obtained with caval subtraction agreed well with those with fluorescent microspheres (mean difference, 4.2%; 95% LOA, ±20.5%). Good consistency was demonstrated between TLBF in humans measured with caval subtraction and direct inflow phase-contrast MR imaging (mean difference, −1.3 mL/min/100 g; 95% LOA, ±23.1 mL/min/100 g). TLBF reproducibility at 7 days was similar between the two methods (95% LOA, ±31.6 mL/min/100 g vs ±29.6 mL/min/100 g).

**Conclusion:**

Caval subtraction phase-contrast MR imaging is a simple and clinically viable method for measuring TLBF and hepatic arterial flow.

*Online supplemental material is available for this article.*

## Abstract (synopsis)

Caval subtraction phase-contrast MR imaging is technically feasible and may offer a reproducible and clinically viable method for measuring total liver blood flow and hepatic arterial flow.

## Introduction

The dual portal venous (PV) and hepatic arterial blood supply to the liver makes it difficult to assess hemodynamic complications of liver disease. To date, invasive methods still remain the standard of reference. Imaging-based hepatic hemodynamic assessment has the potential to yield useful and meaningful biomarkers of portal hypertension and chronic liver disease (1).

Two-dimensional (2D) phase-contrast magnetic resonance (MR) imaging is an established, widely available, and validated method for the noninvasive measurement of large-vessel bulk flow (2–5). Several investigators have used phase-contrast MR imaging to study PVportal vein flow, although few have reported data from the proper hepatic artery (6–9) because evaluation is challenging in routine clinical practice, primarily because of small vessel size, tortuosity, and anatomic variation. Furthermore, low signal-to-noise ratio, vessel orthogonality, partial volume averaging errors, intravoxel phase dispersion, and spatial misregistration can confound estimation of pulsatile flow in small arteries and have impeded both research and clinical use of phase-contrast MR imaging for the measurement of total liver blood flow (TLBF).

Recognizing these challenges, we propose an alternative application of 2Dtwo-dimensional phase-contrast MR imaging to measure TLBFtotal liver blood flow and proper hepatic arterial flow by using caval subtraction. TLBFtotal liver blood flow and hepatic arterial flow fraction could be valuable hemodynamic biomarkers of liver disease. The purpose of this study was to validate caval subtraction 2Dtwo-dimensional phase-contrast MR imaging measurements of TLBFtotal liver blood flow and hepatic arterial fraction in a preclinical animal model and evaluate consistency and reproducibility in humans.

## Materials and Methods

### Background Theory

From the application of the principle of conservation of mass to flow (*Q*), for a fixed tissue volume:
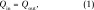
where *Q*_in_ is blood flow into the organ and *Q*_out_ is blood flow out of the organ. On the basis of the anatomic configuration of the liver, the intrahepatic inferior vena cava (IVC) receives blood entirely from the hepatic venous system. Outflow TLBFtotal liver blood flow (*Q*_out_, equivalent to *Q*_TLBF_) can therefore be estimated by measuring bulk flow in the suprahepatic subcardiac portion of the IVCinferior vena cava (*Q*_suprahepatic_) and then subtracting flow from the infrahepatic, suprarenal portion of the IVCinferior vena cava (*Q*_infrahepatic_) (Fig E1a [online]), as follows:
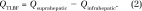
Hepatic arterial flow (*Q*_HA_) can then be estimated by subtracting directly measured PVportal vein flow (*Q*_PV_) from the outflow TLBFtotal liver blood flow (Fig E1b [online], as follows:
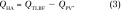


### Preclinical Validation

*Subjects and preparation.—*All experiments were conducted according to the home office guidelines under the U.K. Animals in Scientific Procedures Act (1986) after approval from the Animal Care Ethical Committee of University College London. Experiments were performed between September 12 and October 17, 2013, on healthy male Sprague-Dawley rats (Charles River UK, Margate, England) with normal liver function (weight, 250–300 g).

The study cohort consisted of 15 healthy animals treated with sham laparotomy (*n* = 11) or bile duct ligation (BDL) (*n* = 4). The latter group was included to test the feasibility of performing phase-contrast MR imaging in an animal model of portal hypertension. BDLbile duct ligation and sham surgery were conducted as described previously (10). After recovery, animals were maintained for 4–5 weeks before undergoing the experimental protocol. All procedures were performed by the study coordinator (M.D.C., a radiology research fellow qualified in animal handling with 4 years of experience).

*Standards of reference.—*For rats that underwent sham operation (*n* = 11), laparotomy was performed and a 2-mm transit-time US probe (Transonic Systems, Ithaca, NY) was placed around the PVportal vein. PVportal vein flow readings were obtained with transit-time US after 10–15 minutes, once the animal was stable. Extensive adhesions around the porta hepatis and associated high risk of traumatic vessel injury precluded transit-time US validation in the BDLbile duct ligation group.

After transit-time US measurement, 15-μm polystyrene fluorescent microspheres (FluoSpheres; Life Technologies, Warrington, England) suspended in heparinized saline were administered transcutaneously into the left ventricle over approximately 10 seconds under US guidance (Terason; Teratech, Burlington, Mass) (Fig E2 [online]). Animals were then transferred to the imager for phase-contrast MR imaging. The animal was then sacrificed and organs were explanted for microsphere processing using an adapted protocol (11), as summarized in Appendix E1 (online). To ensure adequate central mixing of microspheres, data exceeding 20% difference in microsphere content between right and left kidneys were excluded.

*Two-dimensional cine phase-contrast MR imaging.—*Temperature was monitored with a rectal probe (SA Instruments, New York, NY), with core body temperature maintained between 36°C and 38°C. Cardiac monitoring was undertaken by using a triple-electrode single-lead system (SA Instruments). Imaging was performed by using a 9.4-T unit (Agilent Technologies, Oxford, England), with sequence parameters listed in the Table. Axial and angled coronal gradient-echo images were used to identify the IVCinferior vena cava and PVportal vein, and phase-contrast image planes were planned to ensure orthogonality with the subcardiac portion of the suprahepatic IVCinferior vena cava, suprarenal portion of the infrahepatic IVCinferior vena cava, and PVportal vein.

**Table:**
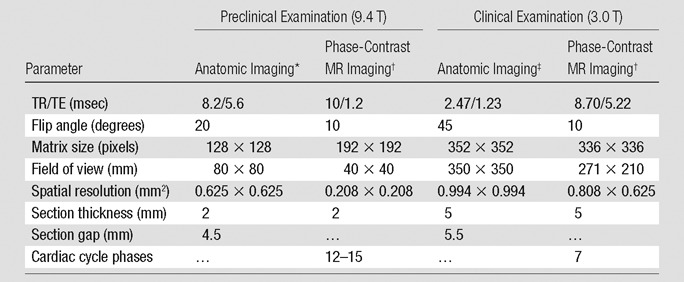
Sequence Parameters

Note.—TR/TE = repetition time/echo time.

*Performed with a gradient-echo sequence.

^†^Performed with a gradient-echo sequence with additional bipolar phase contrast gradients.

^‡^Performed with steady-state free precession.

Cardiac- and respiratory-gated 2Dtwo-dimensional cine phase-contrast MR imaging was performed with 2-mm-thick sections, a 10° flip angle, and a 192 × 192 (frequency encoding × phase encoding) acquisition matrix. On the basis of initial pilot work in four animals (not presented in this study), velocity encoding (*V*_enc_) settings of 33 cm/sec for PVportal vein and infrahepatic IVCinferior vena cava flows and 66 cm/sec for suprahepatic IVCinferior vena cava flows were applied (Table). Phase maps were acquired at each *V*_enc_velocity encoding setting with opposite flow-encoding directions. Correction for background phase errors was achieved by subtracting phase maps with opposing flow-encoding directions, with the assumption that the phase of stationary spins was identical in each image. Acquisition time for each phase-contrast MR imaging measurement was usually less than 10 minutes. Regions of interest were positioned manually by the study coordinator on each vessel for each frame of the cardiac cycle, and flow quantification was performed by using in-house-developed Matlab code (MathWorks, Natick, Mass) on the basis of established algorithms (12). All PVportal vein flow, estimated TLBFtotal liver blood flow, and hepatic arterial flow measurements were normalized to explanted liver weight.

### Human Translation

Two aspects of the caval subtraction technique were then tested in healthy volunteers: *(a)* consistency with directly measured phase-contrast MR imaging PVportal vein and hepatic arterial inflow and *(b)* measurement reproducibility after 7 days.

*Subjects and preparation.—*University College London Ethics Committee approval was obtained, and participants provided informed written consent. Volunteers were recruited by means of advertisement within the university campus and were eligible if they *(a)* had no contraindications to MR imaging, *(b)* were not taking any long-term medication (excluding the oral contraceptive pill), and *(c)* had no documented history of previous liver or gastrointestinal disease. Fourteen volunteers were screened, one of whom was excluded because of claustrophobia. The final cohort consisted of seven men (mean age ± standard deviation, 26.5 years ± 1.36) and six women (mean age, 31.2 years ± 2.62) who underwent imaging between June 14 and July 25, 2013. Participants fasted for 6 hours before MR imaging and avoided caffeinated fluids.

*Two-dimensional cine phase-contrast MR imaging.—*Phase-contrast MR imaging was performed with a 3.0-T unit (Achieva; Philips Healthcare, Best, the Netherlands) and a 16-channel body coil (SENSE XL-Torso, Philips Healthcare). Imaging parameters are given in the Table.

Coronal (upper abdomen), sagittal (abdominal great vessels), and oblique (PVportal vein) breath-hold balanced steady-state free precession images were acquired. Two-dimensional phase-contrast MR imaging with expiratory breath hold and retrospective cardiac gating was planned in two planes by the study coordinator to ensure orthogonality to the target vessel. Studies were performed through the PVportal vein (*V*_enc_velocity encoding = 40 cm/sec), proper hepatic artery (*V*_enc_velocity encoding = 60 cm/sec), infrahepatic IVCinferior vena cava (above the renal veins, below the hepatic IVCinferior vena cava; *V*_enc_velocity encoding = 60 cm/sec), and suprahepatic IVCinferior vena cava (above the hepatic venous inflow, below the right atrial junction; *V*_enc_velocity encoding = 80 cm/sec). Where hepatic arterial anatomy varied (*n* = 2), measurements were made as close as possible to the origin of the hepatic artery. Images were reviewed for aliasing and *V*_enc_velocity encoding settings increased by 20 cm/sec when appropriate. Data were acquired by using the unit’s clinical flow quantification implementation. Phase maps were acquired at each *V*_enc_velocity encoding setting with opposite flow-encoding directions. Correction for background phase errors was achieved by subtracting phase maps with opposing flow-encoding directions, with the assumption that the phase of stationary spins was identical in each image. A local phase-correction filter was also applied to correct for phase errors induced by eddy currents. The acquisition time for each measurement was less than 20 seconds. Each phase-contrast MR imaging study was repeated three times. Flow quantification was performed by using freely available software (Segment; Medviso, Lund, Sweden) and the mean of triplicate measurements used for analysis. Caval subtraction TLBFtotal liver blood flow, PVportal vein flow, hepatic arterial flow, and hepatic arterial fraction were calculated (Eqq [2, 3]) and compared with direct phase-contrast MR imaging of PVportal vein and hepatic arterial inflow.

Liver volume was estimated by using steady-state free precession coronal images with 5-mm-thick sections. Segmentation was performed manually by the study coordinator using software (Amira Resolve RT; Visage Imaging, Berlin, Germany). A tissue density of 1.0 g/mL was assumed (13).

Seven days after the original study, subjects underwent repeat imaging with identical preparation and MR imaging protocol at a comparable time of the day. All analyses and quantification were performed by the study coordinator (M.D.C., with 5 years of experience in abdominal imaging).

### Statistical Analysis

Data normality was confirmed with Kolmogorov-Smirnov testing. All bulk flow measurements obtained at phase-contrast MR imaging were normalized to liver weight or volume. Comparison between measurements derived from caval subtraction phase-contrast MR imaging and standards of reference (transit-time US, microspheres, direct inflow phase-contrast MR imaging) and 7-day reproducibility studies were assessed by using Bland-Altman analysis of agreement, with calculation of 95% limits of agreement (LOA). Coefficients of variation were also calculated and compared by using methods described by Forkman (14). Because of the small number of animals in the sham and BDLbile duct ligation groups that underwent validation with microsphere analysis, validation analysis was pooled across both cohorts and the Mann-Whitney *U* test used for comparison of PVportal vein and relative hepatic arterial flow. Data are expressed as means ± standard errors, and *P* < .05 was indicative of a statistically significant difference.

## Results

### Preclinical Cohort

Across 15 animals, the mean body weight was 451.7 g ± 9.0 and the wet liver mass was 24.7 g ± 2.0. All animals in the BDLbile duct ligation group had evidence of cirrhosis at histopathologic examination.

*Technical feasibility of caval subtraction phase-contrast MR imaging.—*Electrocardiographically and respiratory-gated cine phase-contrast MR imaging flow studies through the cardiac cycle demonstrated physiologic flow profiles through the PVportal vein and infrahepatic and suprahepatic IVCinferior vena cava (Fig E3 [online]).

*Validation of PV and relative hepatic arterial flow.—*Comparing phase-contrast MR imaging versus transit-time US (*n* = 11), the mean difference between PVportal vein flow directly measured with phase-contrast MR imaging (mean, 182.8 mL/min/100 g ± 9.9) and transit-time US (mean, 186.3 mL/min/100 g ± 11.9) was −3.5 mL/min/100 g ± 9.4, with 95% LOAlimits of agreement of ±61.3 mL/min/100 g (Fig 1a). The coefficient of variation for PV flow was similar for transit-time US (21.2%) compared with phase-contrast MR imaging (17.9%) (*F*_10,10_ = 1.38, *P* = .31).

**Figure 1a:**
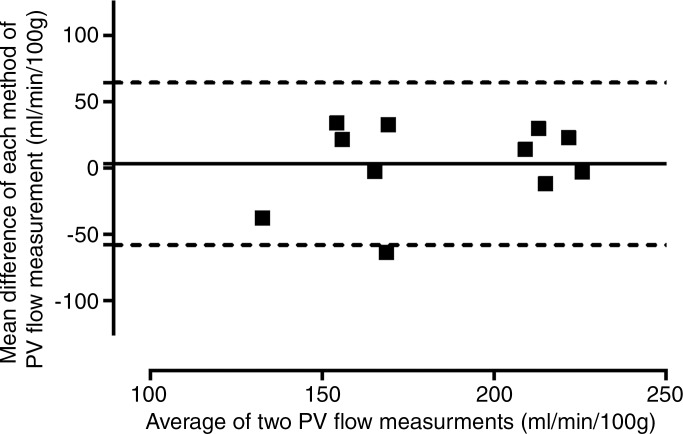
Validation of phase-contrast MR imaging PVportal vein flow and caval subtraction phase-contrast MR imaging–derived hepatic arterial *(HA)* fraction in sham-operated (■) and BDLbile duct ligation (▲) rats. **(a, c)** Bland-Altman plots and **(b, d)** scatterplots show agreement between **(a, b)** PVportal vein flow at phase-contrast MR imaging and transit-time US *(TTUS)* and **(c, d)** hepatic arterial fraction at caval subtraction phase-contrast MR imaging and fluorescent microspheres.

**Figure 1b:**
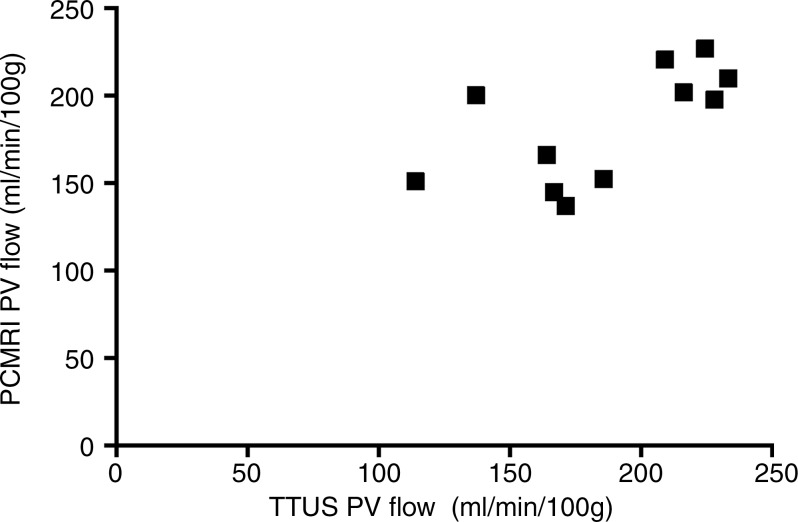
Validation of phase-contrast MR imaging PVportal vein flow and caval subtraction phase-contrast MR imaging–derived hepatic arterial *(HA)* fraction in sham-operated (■) and BDLbile duct ligation (▲) rats. **(a, c)** Bland-Altman plots and **(b, d)** scatterplots show agreement between **(a, b)** PVportal vein flow at phase-contrast MR imaging and transit-time US *(TTUS)* and **(c, d)** hepatic arterial fraction at caval subtraction phase-contrast MR imaging and fluorescent microspheres.

**Figure 1c:**
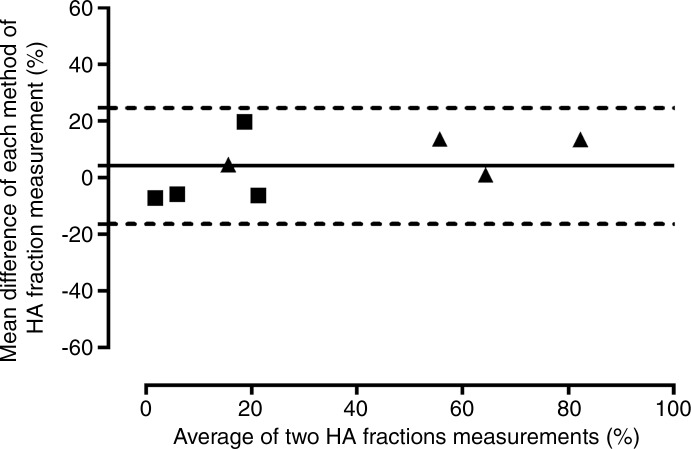
Validation of phase-contrast MR imaging PVportal vein flow and caval subtraction phase-contrast MR imaging–derived hepatic arterial *(HA)* fraction in sham-operated (■) and BDLbile duct ligation (▲) rats. **(a, c)** Bland-Altman plots and **(b, d)** scatterplots show agreement between **(a, b)** PVportal vein flow at phase-contrast MR imaging and transit-time US *(TTUS)* and **(c, d)** hepatic arterial fraction at caval subtraction phase-contrast MR imaging and fluorescent microspheres.

**Figure 1d:**
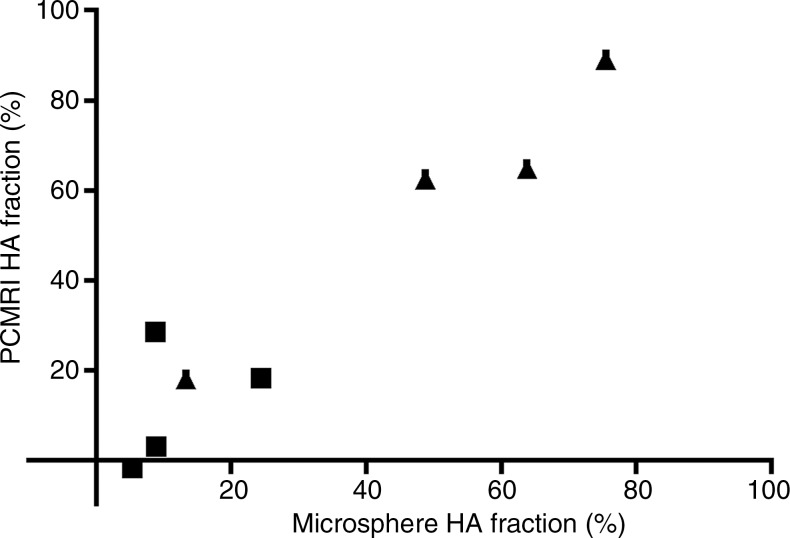
Validation of phase-contrast MR imaging PVportal vein flow and caval subtraction phase-contrast MR imaging–derived hepatic arterial *(HA)* fraction in sham-operated (■) and BDLbile duct ligation (▲) rats. **(a, c)** Bland-Altman plots and **(b, d)** scatterplots show agreement between **(a, b)** PVportal vein flow at phase-contrast MR imaging and transit-time US *(TTUS)* and **(c, d)** hepatic arterial fraction at caval subtraction phase-contrast MR imaging and fluorescent microspheres.

In the comparison of phase-contrast MR imaging with microspheres, seven animals had inadequate central mixing of microspheres and were excluded, leaving eight for analysis (sham, *n* = 4; BDL, *n* = 4). The mean difference between relative hepatic arterial flow derived from phase-contrast MR imaging with caval subtraction (Eq [3]; mean, 35.3% ± 11.6) and that calculated from the microsphere distribution analysis (mean, 31.1% ± 9.8) was 4.2% ± 3.7, with 95% LOAlimits of agreement of ±20.5% (Fig 1c). The coefficient of variation was similar for caval subtraction phase-contrast MR imaging (93.0%) and microsphere analysis (89.2%) (F_7,7_ = 0.95, *P* = .52). Relative hepatic arterial flow was greater and PVportal vein flow was lower in animals with cirrhosis and portal hypertension (relative hepatic arterial flow: 50.3% ± 13.5 in BDLbile duct ligation group vs 11.8% ± 4.3 in sham group; PVportal vein flow: 94.3 mL/min/100 g ± 28.8 in BDLbile duct ligation group vs 167.0 mL/min/100 g ± 20.3 in sham group), but these differences were not statistically significant (hepatic arterial fraction, *P* = .0571; PVportal vein flow, *P* = .200).

### Clinical Cohort

The mean liver volume was 1211.0 mL ± 52.9. Two subjects declined subsequent examination, leaving 11 in the reproducibility cohort.

*Technical feasibility of caval subtraction phase-contrast MR imaging.—*Electrocardiographically and respiratory-gated cine phase-contrast MR imaging flow studies through the cardiac cycle demonstrated physiologic flow profiles through the PVportal vein, infrahepatic IVCinferior vena cava, and suprahepatic IVCinferior vena cava (Fig E4 [online]).

*Caval subtraction phase-contrast MR imaging versus direct phase-contrast MR imaging.—*The mean difference between TLBFtotal liver blood flow measured with caval subtraction phase-contrast MR imaging (mean, 71.1 mL/min/100 g ± 3.3) and that measured with direct phase-contrast MR imaging (sum of PVportal vein and common hepatic arterial flow; mean, 72.5 mL/min/100 g ± 3.3) and between calculated hepatic arterial flow (Eq [3]; mean, 14.0 mL/min/100 g ± 3.5) and direct phase-contrast MR imaging measured hepatic arterial flow (mean, 15.3 mL/min/100 g ± 2.4) was −1.3 mL/min/100 g ± 2.4. The 95% LOAlimits of agreement for caval subtraction versus direct inflow phase-contrast MR imaging were ±23.1 mL/min/100 g for both TLBFtotal liver blood flow and hepatic arterial flow (range, 40.5–100.9 mL/min/100 g and −20.7 to 54.9 mL/min/100 g, respectively) (Fig 2a, 2c). The coefficient of variation for caval subtraction phase-contrast MR imaging TLBFtotal liver blood flow (23.0%) was similar to that for direct inflow TLBFtotal liver blood flow (22.2%) (*F*_23,23_ = 0.94, *P* = .56). Hepatic arterial flow with caval subtraction phase-contrast MR imaging (123.2%) was higher than that with direct phase-contrast MR imaging (75.6%), although this did not reach statistical significance (*F*_23,23_ = 1.68, *P* = .11).

**Figure 2a:**
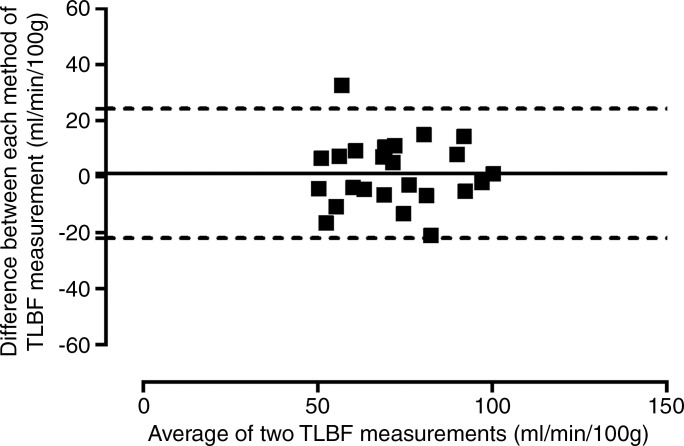
Consistency of caval subtraction phase-contrast MR imaging. Caval subtraction phase-contrast MR imaging TLBFtotal liver blood flow and hepatic arterial flow in healthy volunteers were compared with contemporaneous inflow phase-contrast MR imaging measurements. Data were pooled from baseline and 7-day reproducibility studies. **(a, c)** Bland-Altman plots and **(b, d)** scatterplots show agreement between **(a, b)** TLBFtotal liver blood flow estimated at caval subtraction imaging and that determined at inflow phase-contrast MR imaging and between **(c, d)** hepatic arterial flow estimated at caval subtraction imaging and proper hepatic arterial flow at inflow phase-contrast MR imaging.

**Figure 2b:**
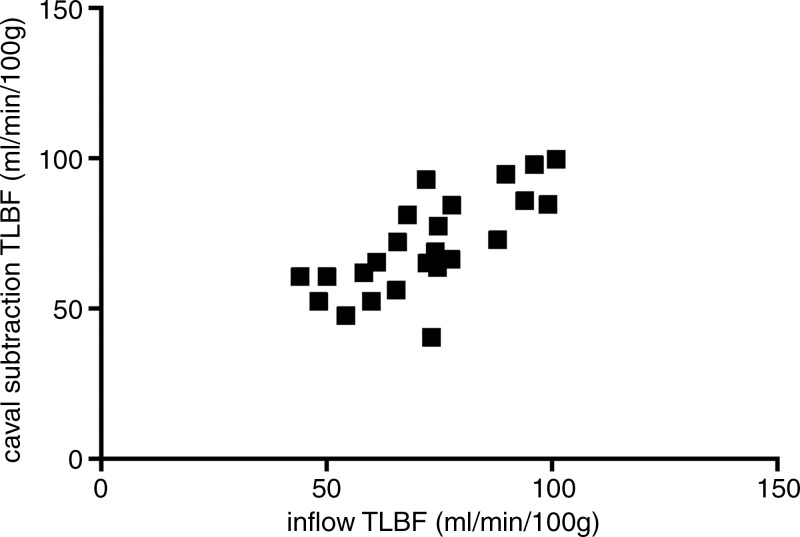
Consistency of caval subtraction phase-contrast MR imaging. Caval subtraction phase-contrast MR imaging TLBFtotal liver blood flow and hepatic arterial flow in healthy volunteers were compared with contemporaneous inflow phase-contrast MR imaging measurements. Data were pooled from baseline and 7-day reproducibility studies. **(a, c)** Bland-Altman plots and **(b, d)** scatterplots show agreement between **(a, b)** TLBFtotal liver blood flow estimated at caval subtraction imaging and that determined at inflow phase-contrast MR imaging and between **(c, d)** hepatic arterial flow estimated at caval subtraction imaging and proper hepatic arterial flow at inflow phase-contrast MR imaging.

**Figure 2c:**
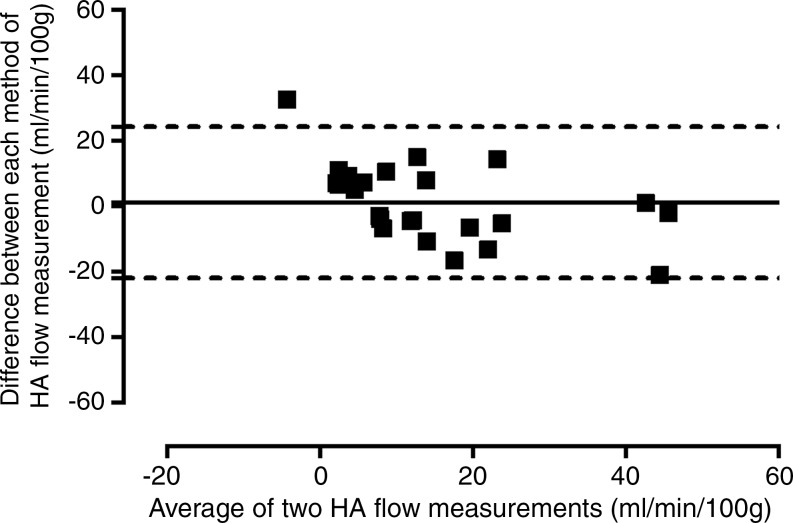
Consistency of caval subtraction phase-contrast MR imaging. Caval subtraction phase-contrast MR imaging TLBFtotal liver blood flow and hepatic arterial flow in healthy volunteers were compared with contemporaneous inflow phase-contrast MR imaging measurements. Data were pooled from baseline and 7-day reproducibility studies. **(a, c)** Bland-Altman plots and **(b, d)** scatterplots show agreement between **(a, b)** TLBFtotal liver blood flow estimated at caval subtraction imaging and that determined at inflow phase-contrast MR imaging and between **(c, d)** hepatic arterial flow estimated at caval subtraction imaging and proper hepatic arterial flow at inflow phase-contrast MR imaging.

**Figure 2d:**
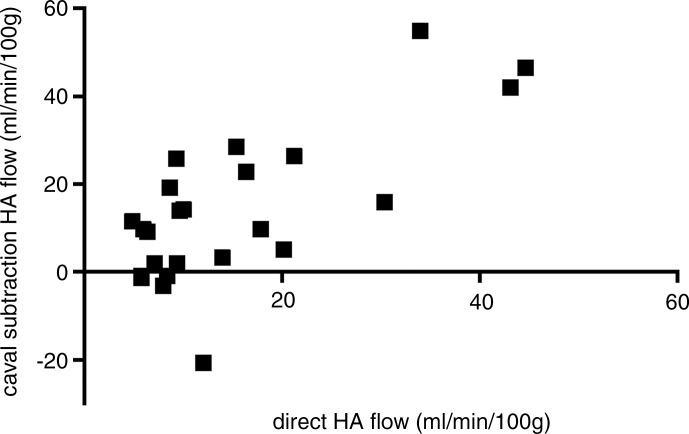
Consistency of caval subtraction phase-contrast MR imaging. Caval subtraction phase-contrast MR imaging TLBFtotal liver blood flow and hepatic arterial flow in healthy volunteers were compared with contemporaneous inflow phase-contrast MR imaging measurements. Data were pooled from baseline and 7-day reproducibility studies. **(a, c)** Bland-Altman plots and **(b, d)** scatterplots show agreement between **(a, b)** TLBFtotal liver blood flow estimated at caval subtraction imaging and that determined at inflow phase-contrast MR imaging and between **(c, d)** hepatic arterial flow estimated at caval subtraction imaging and proper hepatic arterial flow at inflow phase-contrast MR imaging.

*Seven-day reproducibility.—*The mean differences between TLBFtotal liver blood flow measured with caval subtraction phase-contrast MR imaging and hepatic arterial flow measurements obtained 7 days apart were −8.5 mL/min/100 g ± 4.9 and 7.3 mL/min/100 g ± 4.4, respectively. The 95% LOAlimits of agreement were ±31.6 mL/min/100 g for TLBFtotal liver blood flow and ±28.8 mL/min/100 g for hepatic arterial flow. The mean difference between directly measured phase-contrast MR imaging TLBFtotal liver blood flow and hepatic arterial flow measurements obtained 7 days apart were −2.3 mL/min/100 g ± 4.5 and 1.1 mL/min/100 g ± 3.0, respectively. The caval subtraction 95% LOAlimits of agreement were ±31.6 mL/min/100 g for TLBFtotal liver blood flow and ±28.8 mL/min/100 g for hepatic arterial flow. The 95% LOAlimits of agreement were ±29.6 mL/min/100 g for TLBFtotal liver blood flow and ±19.5 mL/min/100 g (range, 4.9–44.6 mL/min/100 g) for hepatic arterial flow.

## Discussion

Accurate assessment of proper hepatic arterial flow (and, consequently, TLBFtotal liver blood flow) with use of phase-contrast MR imaging is challenging in both preclinical and clinical contexts. In rodents, the vessel itself is extremely small—less than 1 mm in diameter (15–17)—which introduces measurement errors, even at high field strengths. Technical challenges have confounded attempts to measure proper hepatic arterial flow in humans with clinical systems (6–9). Adequate signal-to-noise ratio is less of an issue because the vessel is larger, but partial voluming and spatial resolution remain problematic, particularly at 1.5 T (7). Furthermore, frequent anatomic variation (18–20) complicates measurement and requires costly radiologic expertise for planning, which substantially impedes clinical implementation.

We have demonstrated that caval subtraction phase-contrast MR imaging provides a relatively simple strategy for overcoming these limitations in both rodents and humans. We found that phase-contrast MR imaging is technically feasible in animals (even in a cirrhotic BDLbile duct ligation model), and phase-contrast MR imaging PVportal vein flow estimates showed encouraging agreement with directly measured transit-time US flow (21). Thereafter, with the caval subtraction technique, reasonable agreement was obtained for hepatic arterial fraction against a microsphere standard of reference. Unfortunately, quantification of absolute hepatic arterial flow was not undertaken because simultaneous peripheral arterial sampling proved unreliable in pilot experiments.

For human translation, we chose not to use transcutaneous Doppler US measurements of PVportal vein and hepatic arterial flow because reported reproducibility is poor (8,22–24). Instead, direct phase-contrast MR imaging measurements of PVportal vein and hepatic arterial flow were used to test consistency of the caval subtraction phase-contrast MR imaging technique. Good agreement for TLBFtotal liver blood flow was demonstrated between the two methods, and although hepatic arterial flow agreement was less impressive, the level of disagreement was not contingent on measurement value (ie, there was no systematic bias). Hepatic arterial flow measurements suffer from error propagation as inaccuracies in IVCinferior vena cava and PVportal vein flow are summated during subtraction. This can result in nonphysiologic results such as negative estimates of hepatic arterial flow (particularly when true hepatic arterial flow is low, as would be expected in healthy volunteers).

Measurements obtained with caval subtraction phase-contrast MR imaging were, however, reassuringly similar to the direct measurements obtained with inflow phase-contrast MR imaging, although variable for both techniques, likely due to natural variation in vessel flow rates contingent on differing subject hydration, for example.

To place the levels of agreement for caval subtraction technique with standards of reference into clinical context, it is known that TLBFtotal liver blood flow can vary by as much as 58% between health and disease (16,25,26), which is much greater than the expected error range found in the current study.

Our study has limitations. Although hepatic venous pressure gradient and portal venous pressure are clinically useful hepatic hemodynamic parameters for determining both management and prognosis (27), the value of absolute flow parameters in clinical practice remains unclear. Caval subtraction phase-contrast MR imaging may also have limitations in the assessment of some patients with chronic liver disease: The presence of large extrahepatic portosystemic shunts (eg, recanalized umbilical vein or gastric varices), retrograde PVportal vein flow, or venous outflow obstruction (Budd-Chiari syndrome) are likely to compromise simple caval subtraction assessment of TLBFtotal liver blood flow or hepatic arterial flow. In addition, gating to a specific phase of respiration can potentially introduce (systematic) errors, particularly if caval blood flow at different levels is variably influenced by respiration phase, a phenomenon that was not investigated in this study. Finally, studies in anesthetized animals and healthy volunteers represent ideal conditions to test the technique given the compliance with imaging protocols. Studies translating this method into potentially less compliant, unwell patients with chronic liver disease are planned and are necessary to determine the value of caval subtraction phase-contrast MR imaging in clinical practice.

Caval subtraction phase-contrast MR imaging is a simple and rapid technique, amenable to technologist-led phase-contrast MR imaging planning in clinical practice. It could also be used for noninvasive validation of more complex methods such as four-dimensional phase-contrast MR imaging. In summary, we have demonstrated that caval subtraction phase-contrast MR imaging is technically feasible and may offer a reproducible and clinically viable method for measuring TLBFtotal liver blood flow and hepatic arterial flow.

**Advances in Knowledge**

■ In a rodent model, portal venous flow measurement obtained with two-dimensional (2D) phase-contrast MR imaging demonstrates good agreement with invasive transit-time US (mean difference, −3.5 mL/min/100 g; Bland-Altman 95% limits of agreement [LOA], ±61.3 mL/min/100 g).
■ In a rodent model, hepatic arterial fraction measurement obtained with caval subtraction 2Dtwo-dimensional phase-contrast MR imaging demonstrates good agreement with an invasive microsphere standard of reference (mean difference, 4.2%; 95% LOAlimits of agreement, ±20.5%).
■ In human volunteers, total liver blood flow (TLBF) estimated with caval subtraction 2Dtwo-dimensional phase-contrast MR imaging shows good agreement with that calculated from direct inflow phase-contrast MR imaging (mean difference, −1.3 mL/min/100 g; 95% LOAlimits of agreement, ±23.1 mL/min/100 g).
■ In human volunteers, caval subtraction TLBFtotal liver blood flow and directly measured inflow phase-contrast MR imaging TLBFtotal liver blood flow at 7 days were similar (95% LOAlimits of agreement, ±31.6 mL/min/100 g vs ±29.6 mL/min/100 g).
■ Caval subtraction 2Dtwo-dimensional phase-contrast MR imaging is a noninvasive, simple, and rapid technique for measuring total liver and hepatic arterial blood flow.

## APPENDIX

Appendix E1

## SUPPLEMENTAL FIGURES

Figure E1:

Figure E2:

Figure E2:

Figure E2:
