# Supplementary material for: Use of Caval Subtraction 2D Phase-Contrast MR Imaging to Measure Total Liver and Hepatic Arterial Blood Flow: Preclinical Validation and Initial Clinical Translation
Source: Radiology. 2016 May 12;280(3):916–23. doi: 10.1148/radiol.2016151832 (PMC5015842; doi:10.1148/radiol.2016151832)
Supplement: Appendix E1 [file ry151832suppa1.pdf]

©RSNA, 2016

10.1148/radiol.2016151832

Appendix E1

Validation was undertaken by using green microspheres (450/480 nm excitation wavelength). The liver, spleen, gut, and mesentery, along with both kidneys, were weighed and stored in saturated potassium hydroxide solution at 37°C before measurement after 3 weeks. Samples were filtered, centrifuged, and washed successively. After the final washing step, 2-(2-ethoxyethoxy)ethyl acetate (Sigma-Aldrich, Dorset, United Kingdom) was added to release the fluorescent particles. The supernatant was then transferred the next day to a 96-well glass plate for spectrophotometric quantification (FLUOstar Omega; BMG Labtech, Ortenberg, Germany).

Microspheres recovered from the gut, mesentery, and spleen (splanchnic beds) were considered analogous to portal venous circulation (28,29), whereas those recovered from the liver were used for hepatic arterial flow quantification. Hepatic arterial fraction ($\mathrm{HA}_{\%}$) was calculated as follows:

$$\mathrm{HA}_{\%} = \frac{M_{\mathrm{liver}}}{M_{\mathrm{liver}} + M_{\mathrm{gut,\ mesentery}} + M_{\mathrm{spleen}}},$$

where M_{organ} refers to the absolute number of microspheres recovered from the respective organ tissue.

References

28. Houdijk AP, Teerlink T, Visser JJ, van Lambalgen AA, van Leeuwen PA. Arginine deficiency in bile duct-ligated rats after surgery: the role of plasma arginase and gut endotoxin restriction. Gastroenterology 1997;113(4):1375–1383.

29. Houdijk AP, van Lambalgen AA, Thijs LG, van Leeuwen PA. Gut endotoxin restriction improves postoperative hemodynamics in the bile duct-ligated rat. Shock 1998;9(4):282–288.
