# Supplementary figures and images for: Use of Caval Subtraction 2D Phase-Contrast MR Imaging to Measure Total Liver and Hepatic Arterial Blood Flow: Preclinical Validation and Initial Clinical Translation
Source: Radiology. 2016 May 12;280(3):916–23. doi: 10.1148/radiol.2016151832 (PMC5015842; doi:10.1148/radiol.2016151832)

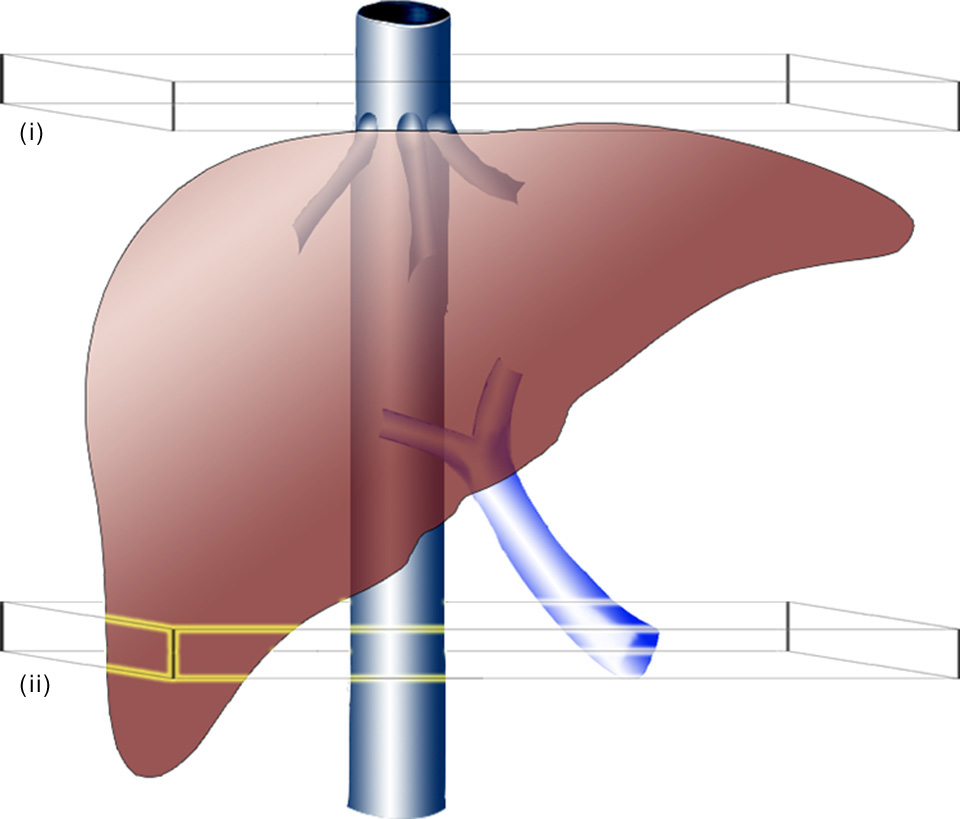

Supplement: Figure E1: [file ry151832suppf1a.jpg]

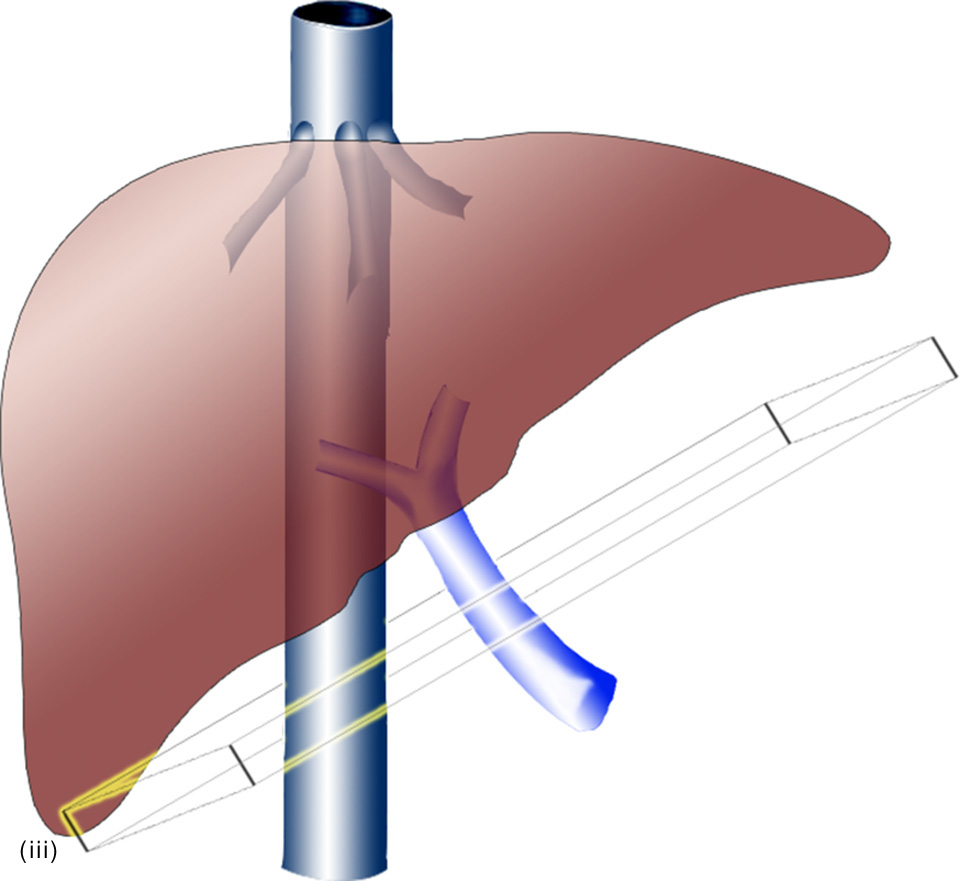

Supplement: Figure E1: [file ry151832suppf1b.jpg]

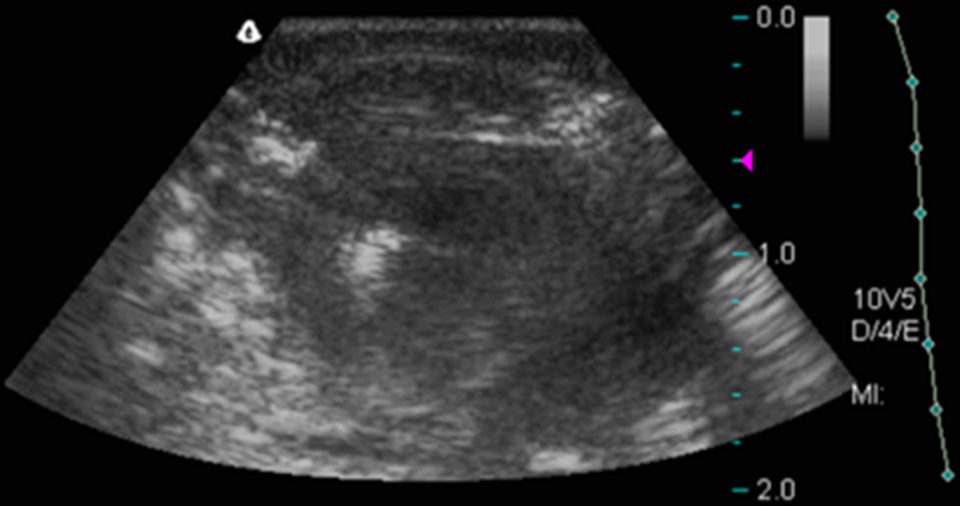

Supplement: Figure E2: [file ry151832suppf2.jpg]

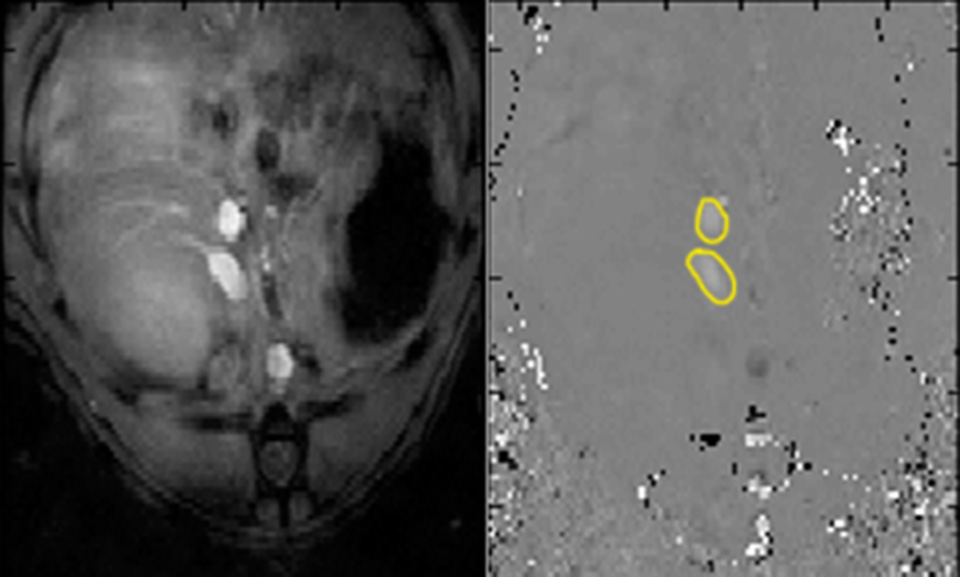

Supplement: Figure E2: [file ry151832suppf3a.jpg]

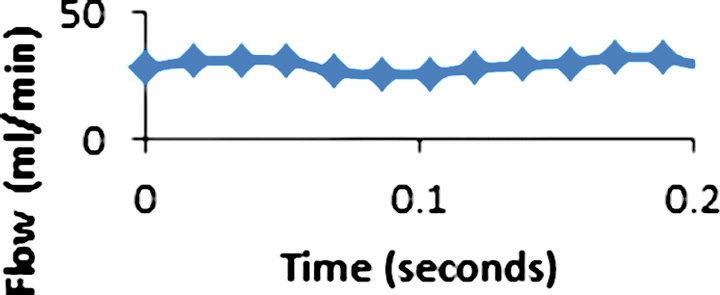

Supplement: Figure E2: [file ry151832suppf3b.jpg]

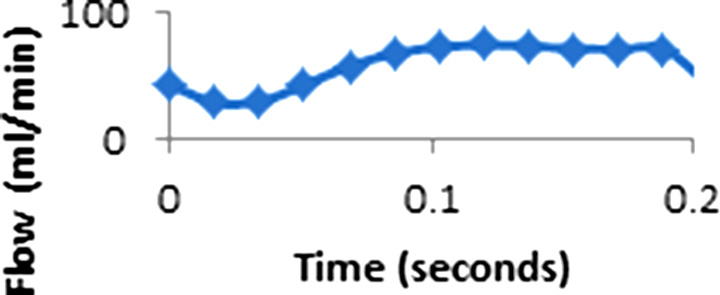

Supplement: Figure E2: [file ry151832suppf3c.jpg]

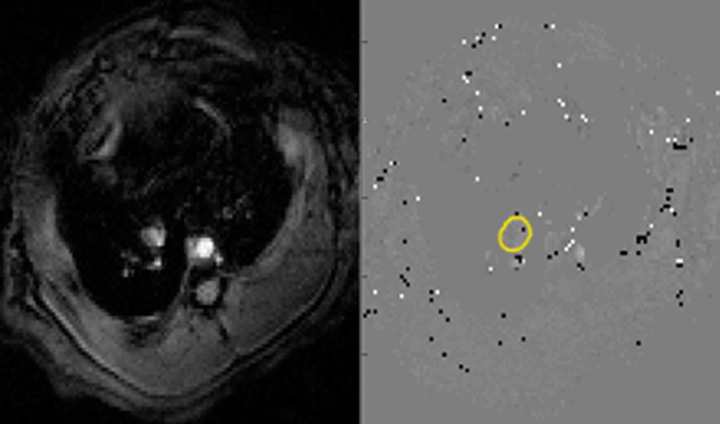

Supplement: Figure E2: [file ry151832suppf3d.jpg]

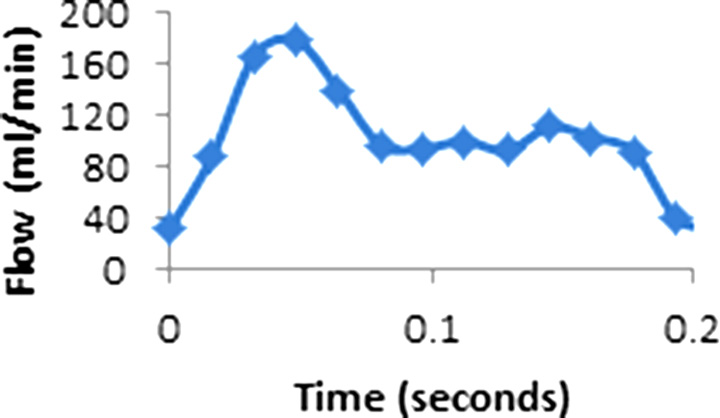

Supplement: Figure E2: [file ry151832suppf3e.jpg]

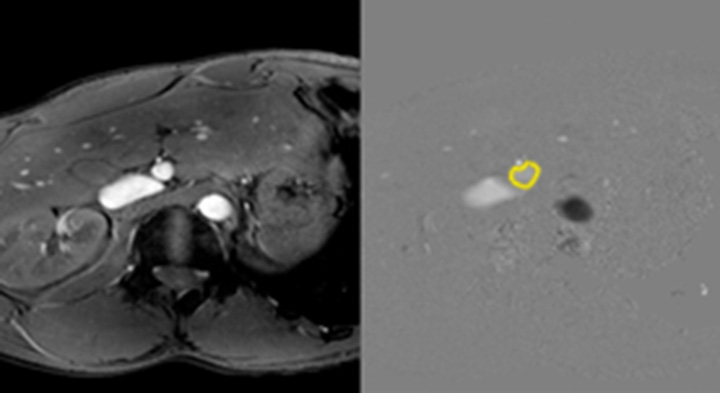

Supplement: Figure E2: [file ry151832suppf4a.jpg]

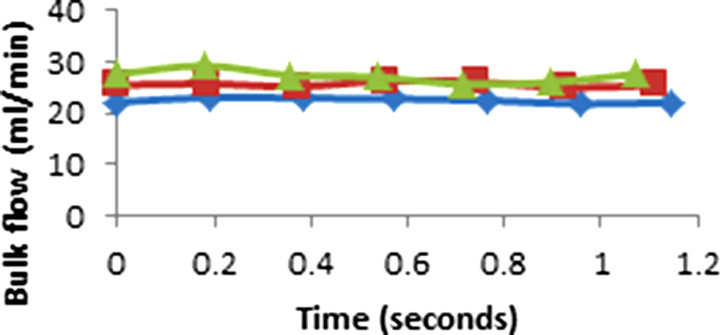

Supplement: Figure E2: [file ry151832suppf4b.jpg]

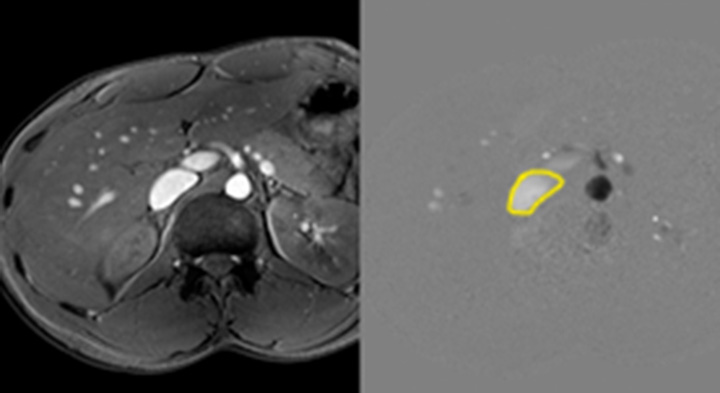

Supplement: Figure E2: [file ry151832suppf4c.jpg]

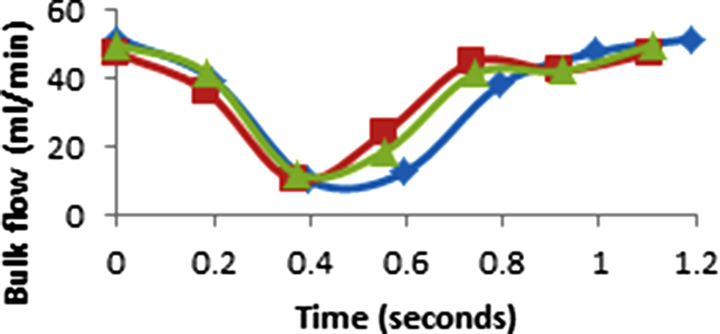

Supplement: Figure E2: [file ry151832suppf4d.jpg]

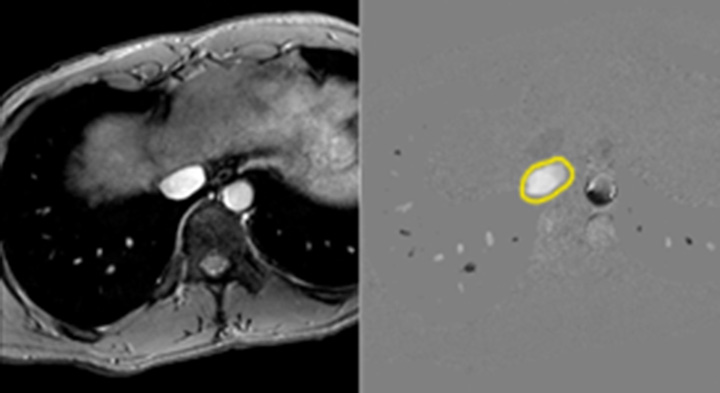

Supplement: Figure E2: [file ry151832suppf4e.jpg]

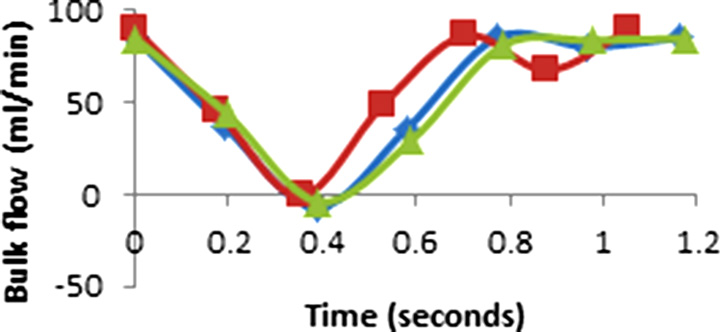

Supplement: Figure E2: [file ry151832suppf4f.jpg]

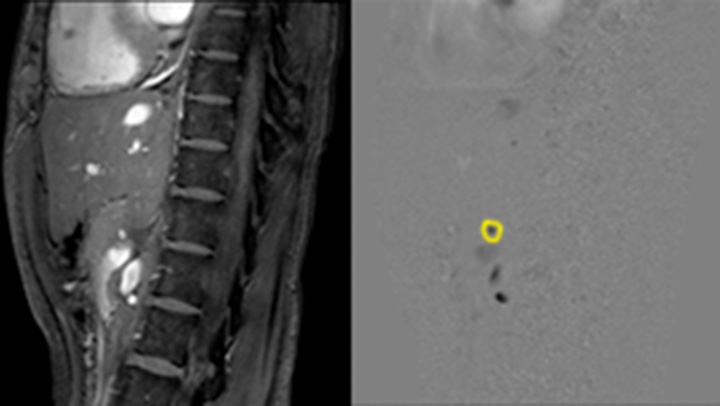

Supplement: Figure E2: [file ry151832suppf4g.jpg]

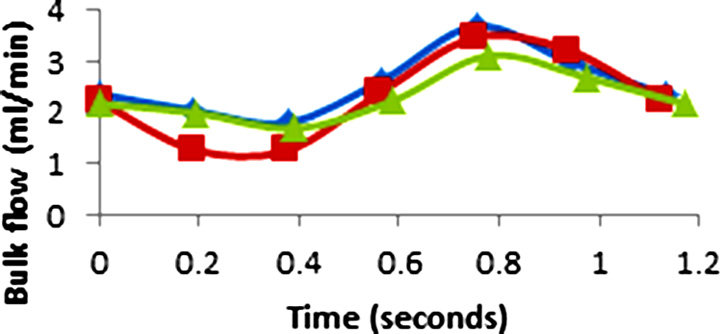

Supplement: Figure E2: [file ry151832suppf4h.jpg]
